# Speeding up the recovery of coastal habitats through management interventions that address constraints on dispersal and recruitment

**DOI:** 10.1098/rspb.2024.1065

**Published:** 2024-07-24

**Authors:** Christopher J. Brown, Max D. Campbell, Catherine J. Collier, Mischa P. Turschwell, Megan I. Saunders, Rod M. Connolly

**Affiliations:** ^1^ Institute for Marine and Antarctic Studies, University of Tasmania, Hobart, Tasmania 7001, Australia; ^2^ Coastal and Marine Research Centre, School of Environment and Science, Australian Rivers Institute, Griffith University, Gold Coast, Queensland, Australia; ^3^ Centre for Tropical Water and Aquatic Ecosystem Research, James Cook University, Cairns, Queensland 4870, Australia; ^4^ CSIRO Environment, Hobart, Tasmania 7001, Australia

**Keywords:** connectivity, multiple stressors, seagrass meadow, population dynamics, Zostera, recovery

## Abstract

Plans for habitat restoration will benefit from predictions of timescales for recovery. Theoretical models have been a powerful tool for informing practical guidelines in planning marine protected areas, suggesting restoration planning could also benefit from a theoretical framework. We developed a model that can predict recovery times following restoration action, under dispersal, recruitment and connectivity constraints. We apply the model to a case study of seagrass restoration and find recovery times following restoration action can vary greatly, from <1 to >20 years. The model also shows how recovery can be accelerated when restoration actions are matched to the constraints on recovery. For example, spreading of propagules can be used when connectivity is the critical restriction. The recovery constraints we articulated mathematically also apply to the restoration of coral reefs, mangroves, saltmarsh, shellfish reefs and macroalgal forests, so our model provides a general framework for choosing restoration actions that accelerate coastal habitat recovery.

## 1. Introduction

Restoration is high on the agenda for managers of structured coastal habitats including seagrass meadows, coral reefs, macroalgal forests, mangrove forests, saltmarsh and shellfish reefs. Restoration is a priority because they supply important ecosystem services [1], which can be recovered through interventions that regrow the habitat forming organisms. Coastal habitats are prone to catastrophic disturbances, like diseases, storms, heatwaves and floods that have the potential to eliminate the habitat from an entire region [2–4] or, more commonly, eliminate a patch of habitat nested within interconnected meta-populations [5–7]. Dispersal of propagules from remnant patches can sometimes facilitate recovery of affected patches [5–7], other times assistance from restoration efforts is necessary [8]. Restoration success is also mixed. Coastal habitats can recover rapidly when conditions are suitable [9], but recovery also commonly fails. For example, seagrass meadows in Australia recovered within 5 years of flood disturbance [4], whereas some meadows on the East Coast of the USA have not recovered from a wasting disease that occurred decades ago [10]. When management actions fail to promote recovery, communities and funders can lose trust in restoration, hindering future recovery attempts [11,12]. Therefore, managers need guidelines for recovery times so that they can identify the timescale for recovery under natural processes or with restoration intervention [13].

Restoration ecology is a practical field drawing on a substantial empirical base to estimate management-relevant quantities, like recovery times and rates of success [9,14,15]. However, the ability to use empirical results to predict outcomes for new restoration sites is limited by context-dependence [16]. Combining theoretical models with empirical data can help identify generalities that facilitate knowledge transfer to new restoration sites [17]. Marine protected area planning is a discipline that has already benefited from a well-developed theoretical framework. Protected area modelling has provided key insights for practice including the role that connectivity plays in success or failure of fish recovery [18] and recovery timelines [19]. The theory and practice in marine protected area planning could be applied to progress theory for restoration ecology. For example, network analysis methods and meta-population modelling tools developed for marine protected areas [18,20] are now being applied to identifying sites for restoration of specific coastal habitat types [6,8]. Protected area models are now also informing restoration guidelines for specific coastal habitats [21]. This parallel between fields suggest we should look to lessons from marine protected area modelling to address the issue of predicting recovery times in restoration ecology.

Marine protected area planning has a well-developed mathematical theory for modelling population dynamics [18,20]. A key result emerging from theoretical studies of marine protected areas is that population dynamics are sensitive to differing assumptions about dispersal, survival and recruitment [22,23]. Habitat patches that appear strongly connected through larval dispersal may only be weakly connected once we consider population dynamics [18,24]. For example, predicted recovery times of overfished areas outside marine reserves vary depending on whether models assume density-dependent survival of dispersing larvae or of post-settlement juveniles [23]. For restoration, therefore, there is a need to synthesize empirical knowledge of coastal habitats into dynamic models of populations to predict how quickly coastal habitats will recover from disturbances, provide guidance on what pre- and post-dispersal constraints are important, and ultimately identify management interventions that will enhance recovery [8].

Here, we develop a model for recovery times of coastal habitats. We consider constraints on the recovery of coastal habitats that are common to all coastal habitat types. The overall objectives of this study were to: (i) estimate how dispersal, recruitment and growth constraints affect recovery times of an extinct habitat patch, (ii) assess how different management interventions influence recovery times, and (iii) explore the sensitivity of recovery times to dispersal and recruitment parameters. Our model is general for any coastal habitat species. Here, we parameterized the model for seagrass meadows of the coastal Great Barrier Reef to provide a tangible example of how the model can inform restoration guidelines. Seagrass meadows, like all coastal habitats, have high value for ecosystem services [25], occur in networks of connected patches [6], are threatened by human stressors globally [26,27], but can recover rapidly when conditions are suitable [9]. Individual meadows can be lost owing to disease, floods or sediment erosion, and long-distance dispersal of propagules (seeds, fruits, viviparous seedlings and specialized shoots) is important for the recovery of these disturbed meadows [28,29]. The Great Barrier Reef represents a good case study for restoration because there are major seagrass restoration efforts that are trialling a variety of management interventions, including catchment actions for water quality improvement and planting [30,31]. Great Barrier Reef seagrass is globally significant, being recognized, for example through its inclusion in the World Heritage Area [30]. The Reef’s seagrass meadows continue to be exposed to major disturbance events, so restoration planning will need to account for heatwaves, storm and flood events [4,6].

## 2. Methods

### (a) General model of habitat recovery

The processes included in the model were chosen to represent any coastal habitat including seagrass meadows, coral reefs, macroalgal forests, mangrove forests, saltmarsh and shellfish reefs. Dominant features of these habitats that were represented in the model include: multiple connected habitat patches each with its own propagule production, a disturbance (e.g. an extreme event or long-term exposure to pollution) that eliminates the population in one patch, interannual variability in propagule supply to the disturbed patch, interannual variability in propagule recruitment at the disturbed patch and environment and density dependent population growth within each patch (electronic supplementary material, tables S1-2 provide examples for application of the model to each habitat type with supporting literature).

We modelled a situation where there was one disturbed (no habitat) patch that was connected to one or more remnant patches that were potential propagule sources. We assumed the disturbed patch had no remnant habitat or seed bank. Propagule supply from each remnant patch was assumed to be proportional to biomass of the habitat in each patch.

**Figure 1.**
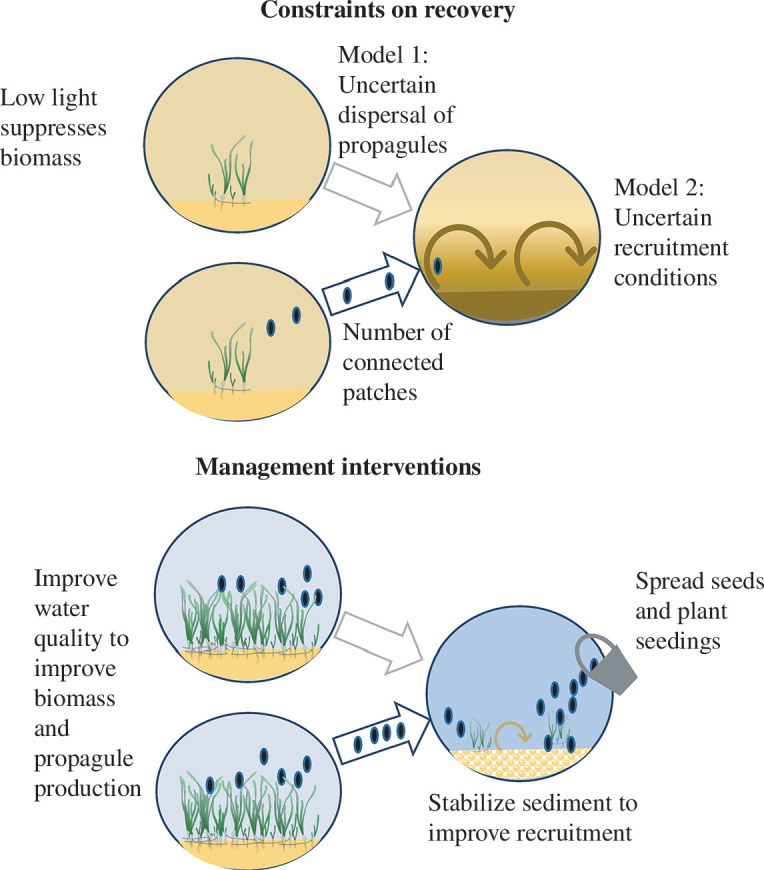
Conceptual diagram of the recovery processes in the model and management intervention options, using seagrass meadows that face chronic stress from low light as an example.

We propose two models of processes that cause uncertainty in the recolonization and recovery time of the disturbed patches (figure 1). In the first model, the chance of propagules reaching the disturbed patch was stochastic, but once propagules reached the patch they recruited 100% of the time (uncertain dispersal model). Uncertain dispersal represents a situation where a disturbed patch receives intermittent supply of propagules from remnant patches because of temporal variability in winds and currents [32].

The second model assumed propagule dispersal to disturbed patches was deterministic, but recruitment of propagules at disturbed patches was stochastic (uncertain recruitment model). Uncertain recruitment can occur when conditions are unsuitable for recruitment, such as sediments or rubble that are too mobile for settlement of propagules [33,34] or resuspension of sediment that reduces light [35] (electronic supplementary material, table S1).

The uncertain dispersal model describes the annual probability of recolonization given the biomass of each remnant patch:


$$p_{D}{\left(B\right)}_{i}\;=1-\prod_{j\;=1}^{n}\left(1\;-\frac{L_{max}}{1\;+\;\;\exp\left(-\nu \left(Q_{ij}B_{j}\;-\;\tau \right)\right)\;}\right),$$

where *n* is the number of remnant patches. The curve had three parameters. The first parameter, *L*_max_ was the maximum annual probability of propagules dispersing from a source meadow to the disturbed meadow. *L*_max_ increased the overall probability of a propagule dispersing each year, so we term it the dispersal suitability parameter. The second, τ was the meadow biomass at which the dispersal probability was half of $L_{max}$ . τ shifts the probability curve to higher biomasses, so we termed it the productivity parameter, because lower values represent greater production of propagules per unit biomass of patch. The third parameter, ν , controlled the steepness of the biomass–dispersal probability curve. Higher values created a threshold between low biomasses with zero probability of propagule dispersal and high biomasses with maximal dispersal probability, whereas lower values created a gradient of dispersal probability from low to high biomass. We termed this parameter the threshold parameter (full details of the model are in the supplemental methods in the electronic supplementary material). Q is a symmetric matrix of connections between patches, where a ‘1’ indicates a connection and ‘0’ indicates no connection. The probability of propagule dispersal to the disturbed meadow increased with the number of connected meadow, and it is bounded by one, at which the chance of dispersal is certain.

The uncertain recruitment model was described by a curve relating the summed biomass of all remnant patch to the probability of recruitment of at least one propagule at the disturbed patch:


$$p_{R}{\left(B\right)}_{i}=\frac{R_{max}}{1\;+exp\left(-\nu {\left(QB\right)}_{i}\;-\;\tau \right)},$$

where *R*_max_ was the maximum annual probability of recruitment when propagules were saturating, which we termed the recruitment suitability parameter. τ was the summed biomass of supply meadows at which the recruitment probability was half of *R*_max_ and ν was the steepness. Under this model, more connected remnant patches meant more propagules that could potentially recruit at the disturbed patch and with high connectivity recolonization would be limited by the recruitment suitability.

We ran all analyses with parameters that represented two seagrass species of *Halodule uninervis* and *Zostera muelleri*. Both are widespread seagrass taxa and several *Zostera* species are currently the subject of restoration attempts [36,37]. To model seagrass growth, we used the formulation of the logistic growth model that represented the effects of light on seagrass growth [38]. Any growth model could be used in its place, and we have suggested how this could be done for other habitat types (electronic supplementary material, table S2). In our model, seagrass biomass grows deterministically towards an equilibrium biomass (see electronic supplementary material, table S3 for species parameters). Seagrass growth at high density was assumed to be limited by self-shading [39]. Growth and maximal density were also impacted by light and temperature conditions. Growth parameters for the seagrass species were taken from the literature or calibrated to values that met *a priori* expectations for recovery from the literature (electronic supplementary material, table S3).

Values for the productivity parameter ( τ ) and the threshold parameter ( ν) are unavailable for seagrass, so we estimated their ranges based on *a priori* expectations. If τ is too much larger than *B*_max_ and ν is too steep (e.g. >0.1), then there will never be any recruitment, because the biomass threshold for seed production is greater than *B*_max_. If ν is too small (e.g. <0.001), the relationship between seed production and recolonization probability will be unrealistically flat. Therefore, we used values intermediate between these extremes. Further, field studies suggest recovery rates for the seagrass species we have studied should be within decades (conditions permitting recovery) [4,40]. These field estimates help us further refine the values of unknown parameters to be within realistic ranges. For a single meadow at *B*_max_ and our target probability for the restoration success rate (electronic supplementary material, table S3), the chosen values reflect a probability of recolonization of 0.24 (64% of *L*_max_ or *R*_max_) for *Zostera* and a probability of recolonization of 0.32 (84% of *L*_max_ or *R*_max_) for *Halodule*. To further explore the importance of these parameters, we looked at the sensitivity of recovery time and the best management action.

We were able to determine the exact solutions for the mean and variability of the recovery times (see electronic supplementary material). The recovery times of these models were exponentially distributed with different rates $\lambda_{S}$ and $\lambda_{R}$ , plus a translation by a constant determined by the rate of biomass growth, which was dependent on light.

### (b) Aim 1: constraints on recovery times

We explored how the modelled recovery time relates to factorial combinations of chronic stress caused by low light (three levels), number of connected patches acting as propagule sources (1–10, poorly through to well connected) and source of uncertainty in recolonization (uncertain dispersal or uncertain recruitment).

We predicted how long it takes the disturbed patch to recover to >50% of its equilibrium biomass (i.e. the maximal biomass given the light level). Fifty per cent recovery is a useful target because changes of that magnitude are detectable with little error [41] and it is asufficient cover to slow current velocities and trap sediment [35]. We assumed that patch size, and therefore growth rates and equilibrium biomass, was the same in all patches. Propagule production was assumed to increase linearly with patch biomass and we assumed there was no seedbank in the disturbed patch (*Zostera* and *Halodule* seeds are short-lived). Recovery under the two recolonization models was predicted for different levels of low-light stress. Low-light stress is caused by declining water quality and increasing turbidity, and is an issue for many coastal habitats [5,6]. Each level of light stress was applied equally to all patches, therefore all non-disturbed patches were at the same equilibrium biomass and had the same propagule production rate. The magnitude of light stress was parameterized from field studies, where stressed meadows grew under 50% of normal light levels, and severely stressed under 25% of normal levels (Supplemental text in electronic supplementary material).

In certain circumstances, empirical studies show that recovery may never happen without intervention because dispersal and/or recruitment constraints are absolute [34,42] and recruitment failure is maintained by alternative stable states [33]. Such situations would be modelled with a recolonization probability = 0. Given results from such a scenario are trivial in practice (i.e. management action to improve recruitment is essential), we chose to model situations where natural recovery was plausible, if unlikely.

### (c) Aim 2: management interventions

We modelled three ways that management could intervene to improve recovery. First, management could spread propagules [43] or plant seedlings [37]. This action was modelled by assuming propagule supply was saturated at the disturbed patch. Second, management could stabilize recruitment conditions, such as using biodegradable structures to stabilize sediment to facilitate seagrass regrowth [44]. This action was modelled by assuming certain recruitment of available propagules. Finally, management could act to mitigate the chronic stressor, such as taking actions in catchments to improve coastal light [45]. Light increases were modelled by comparing simulations with and without low-light stress.

### (d) Aim 3: sensitivity analysis

The parameters of the recruitment and dispersal functions were not well described in the literature, so we tested the sensitivity of conclusions to parameters of the recolonization functions ranging from a four-times reduction to a four-times increase in each parameter value. An eight-times change in parameter values was sufficient to span the range of biologically reasonable parameter values.

## 3. Results

### (a) Aim 1: constraints on recovery times

**Figure 2.**
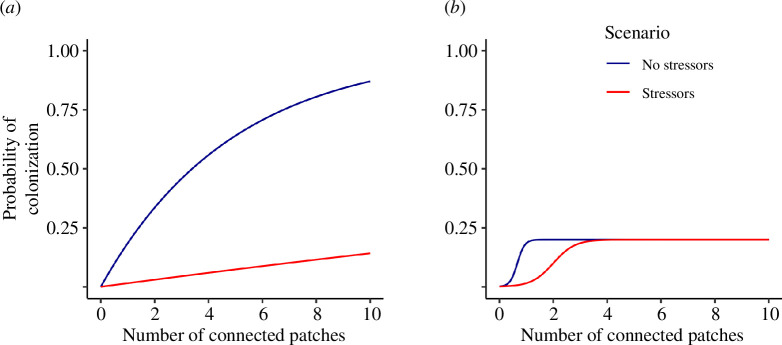
The probability of a disturbed meadow being recolonized changes with increasing numbers of connections to undisturbed patches (degree) under uncertain dispersal (*a*) and uncertain recruitment (*b*). Both seagrass species had the same colonization functions. Note the asymptote in the uncertain recruitment model (*b*) could be <1, whereas the asymptote for the uncertain dispersal model (*a*) was always = 1.

Recolonization of the disturbed patch was more likely with increasing numbers of connections to source meadows (figure 2). The probability of recolonization increased continuously with greater connections under the uncertain dispersal model, but plateaued at the maximum annual probability of recruitment (*L*_max_) under uncertain recruitment (figure 2).

A disturbed *Halodule* patch recovered faster than a *Zostera* patch on average when there was only one connected meadow, whereas *Zostera* recovered faster when there were >2 patches connected. Recovery times were highly variables for both species, with *Zostera* having greater variability than *Halodule*. For example, the median recovery time for *Zostera* was 4.8 years when connected to one patch, but the 95% quantiles spanned 0.4–20.4 years.

**Figure 3.**
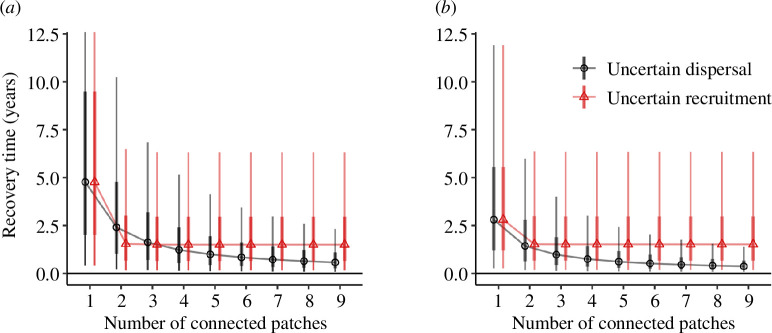
Recovery times for (*a*) *Zostera* and (*b*) *Halodule* under the two recolonization models. Thick bars show 75% quantiles, thin bars show 95% quantiles. Points are offset slightly on the *x*-axis to avoid overplotting. Note that the *y*-axis was cropped at 12.5 years for clarity, the 95% quantile for recovery time for (*a*) and one connected patch was 20.4 years.

Under uncertain dispersal, recovery times reduced continuously as the number of connected patches increased (figure 3*a*), because recovery was limited only by the probability of receiving propagules (figure 2*a*). Greater numbers of connected patches also continuously reduced the variability in recovery time. For example, at three connected patches for *Zostera* the 95% quantiles for recovery times were 0.17–6.8 years, whereas at 10 connected patches, they were 0.09–2.1 years.

Under uncertain recruitment, recovery time plateaued at a minimum at only two connected patches (figure 3), because recovery was then limited by suitable recruitment conditions rather than propagule supply (i.e. the asymptote in figure 2*b*). Variability in recovery time for the uncertain recruitment model remained high as the number of connected patches increased and ranged from 0.16 to 6.5 years (figure 3).

**Figure 4.**
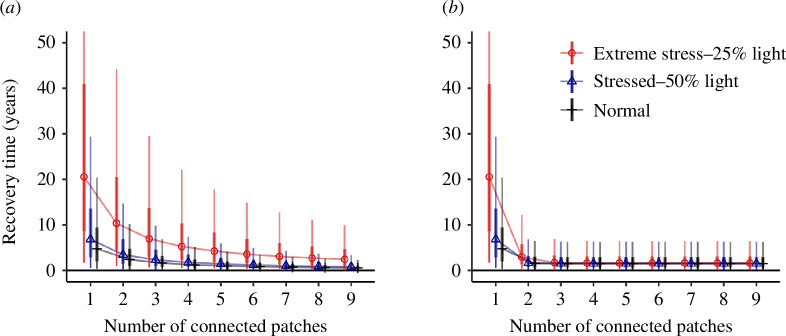
Recovery time under scenarios of low-light stress for *Zostera* and (*a*) uncertain dispersal and (*b*) uncertain recruitment. Note *y*-axis is cropped at 50 years for clarity, but quantiles recovery times for one connected patch exceed 50 years. Results for *Halodule* were similar.

Low-light stress increased recovery times, and variability in recovery time, relative to the control treatments with normal light (figure 4). The median recovery time for a 50% light scenario was within 2 years of the normal light. With extreme low-light stress (25% of normal) at the lowest level of connectivity, recovery times jumped to be >15 years greater than for normal light. Variability in recovery times was greater with low light than with normal light. The impact of low light on recovery time was greatest for low connectivity to propagule sources and negligible for high connectivity to propagule sources.

Under uncertain dispersal, the difference between the normal light and low-light treatments reduced continuously as the number of propagule sources was increased, but the stressor scenarios were never the same (figure 4*a*). Under uncertain recruitment, all stressor scenarios had practically the same distribution of recovery times with three or more propagule sources (figure 4*b*), because the maximum recolonization probability was almost reached by all scenarios at this point.

### (b) Aim 2: management interventions

**Figure 5.**
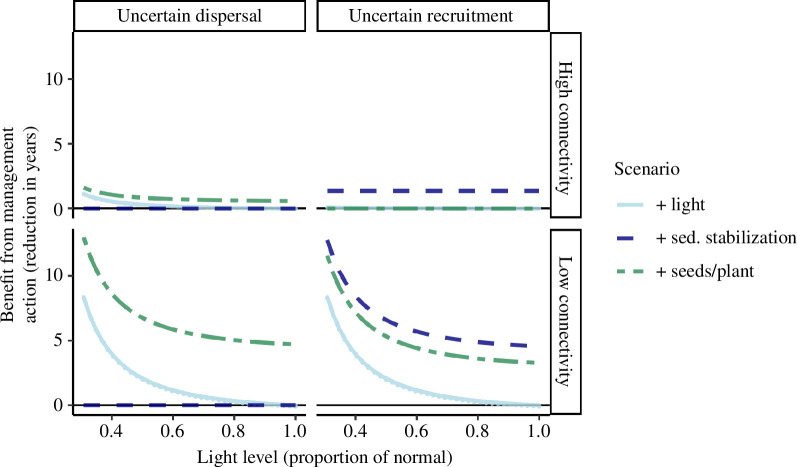
Years of recovery time saved for *Z. muelleri* when comparing different management scenarios to the baseline scenario of no management, assuming the dispersal limited and recruitment limited models (columns) and low (one connected meadow) versus high (eight connected meadows) connectivity to propagule sources (rows). Lines show median gain in recovery times.

Management interventions could speed up the median recovery time the most when there were few connected patches and light was low (by up to 13 years for light at 25% of normal and low connectivity), and had less benefit when there were many connected patches (<2 years) or light was close to normal (figure 5). For uncertain dispersal, dispersing seeds or planting had the greatest benefit, speeding median recovery times by up to 13 years at low light levels, whereas stabilizing recruitment conditions had no effect on recovery times. For uncertain recruitment, stabilizing recruitment conditions was the most effective management intervention, but seeding or planting was nearly as beneficial if connectivity was low (under normal light a median reduction of 3.3 years versus 4.6 years).

Improving benthic light sped recovery times when there were few connected patches regardless of whether there was uncertain recruitment or dispersal. *Halodule* spp*.* (electronic supplementary material, figure S2) had slightly smaller reductions in recovery time than for *Z. muelleri* (figure 5).

### (c) Aim 3: sensitivity of recovery times to recruitment and dispersal parameters

Recovery time was most sensitive to the biomass productivity parameter ( τ , electronic supplementary material, figure S3). Increasing the productivity parameter meant the supply of propagules was lower at a given level of connectivity. There was an exponential increase in recovery times above a τ of 500 g dry wt m^–2^. An exception was the uncertain recruitment model with many connected patches—there was sufficient supply of propagules to saturate recruitment even at high values of the productivity parameter. Recovery time increased exponentially for increasing values of the threshold parameter (note near linear trends in electronic supplementary material, figure S3*c,d*).

We studied the sensitivity of the benefit of management actions to τ , the most important parameter (electronic supplementary material, figure S4). The management action with the fastest recovery time was the same regardless of the biomass productivity parameter. The reduction in recovery time was sensitive to the parameter under low connectivity for: the uncertain recruitment model with sediment stabilization and, the uncertain dispersal model with seeds or planting. Under these management scenarios, recovery was exponentially faster for greater values of the biomass productivity parameter.

## 4. Discussion

Models enable synthesis of recovery processes known from empirical studies, including dispersal, recruitment and propagule production dynamics. Integrating these processes into a single model can help identify the relative importance of these processes and identify priorities for how management can act when faced with uncertain recovery dynamics [8]. Meta-population modelling has focused on representing networks of connected populations, and then studying the resilience of networks to disturbance [6,46]. Network analysis can reveal critical populations that if lost would have a large impact on population connectivity and presumably abundance within patches. However, network models generally do not represent population dynamics, so do not predict timescales of recovery.

Our model predicted that a greater supply of propagules reduced recovery time of seagrass meadows, and reduced the variability in expected recovery times. Recovery times without management actions were likely to be greater than 5 years for *Zostera* and possibly greater than a decade, even for well-connected meadows. The timespans for recovery we predicted are within range of field observations of recovery, which vary but can be lengthy [4,40]. Our prediction of high variability in recovery times may also explain why some seagrass meadows have been observed not to recover from disturbances, even though conditions seem suitable [12]. The predictions of recovery time could be used to inform manager and community expectations. Timelines of management funding are typically 2–4 years [14], but recovery may take considerably longer. Many coastal restoration programmes involve community members [47], so if expectations for recovery times are not clearly communicated, community members may become disengaged with restoration programs.

The timescale of recovery was sensitive to the process we assumed was causing uncertainty in recolonization, either dispersal or recruitment. When connectivity to propagule sources was low, both the uncertain dispersal and uncertain recruitment models predicted median recovery times longer than 3 years, but with high variability in recovery times such that recovery in >10 years was as likely as recovery in 1 year. When the supply of propagules was high, recovery was almost certain within 1–2 years for the uncertain dispersal model, but there was still a possibility of recovery taking >5 years with the uncertain recruitment model. In practical terms, this result means that in regions with many remnant habitat patches and that are hydrologically well-mixed, recruitment limitation will be the main cause of lengthy recovery times. The model also suggests that uncertain dispersal or recruitment conditions cannot be distinguished as causes of non-recovery of patches that are isolated from propagule sources, because recovery times will be similar. When the cause cannot be distinguished, further information such as models of environmental suitability [42] or experiments with both planted seeds and propagule stabilization [48] are needed to identify the primary cause of non-recovery.

Our model can inform how management prioritizes resources for restoration. Marine restoration has had high rates of habitats failing to regrow in the past [14]. Many restoration attempts have been experimental [49] and the restoration approach may not have matched the ecological constraints on recovery. Managers now have many options for techniques for restoration of coastal habitats [44,49]. Our model can be developed with basic information on habitat growth, such as growth measured as per cent cover or biomass change over time. Colonization processes are also important, and information required for the model includes estimates of propagule production at source patches, dispersal among patches and probability that propagules that successfully disperse to the disturbed site then go onto recruit. Colonization information may be more challenging to obtain, so expert elicitation could be used to put a bounds on the dispersal and recruitment parameters, to then estimate best and worst case recovery times. Building the model provides a systematic process for experts to consider constraints on recovery, and appropriate restoration responses.

Our results, which were designed to generalize across common seagrass species, predicted that if dispersal was uncertain, then seeding or planting propagules will speed recovery the most. If recruitment was uncertain, efforts to stabilize sediment will promote recovery the most, but seeding or planting were similarly as beneficial if connectivity to remnant meadows was also low. This result is indicative of the model’s realism, because it is consistent with our understanding of why seagrass restoration does and does not work in different places [44]. The results support prioritizing seeding or planting for meadows that are isolated from remnant meadows, and also in well-connected meadows that receive an intermittent supply of propagules because of hydrological variability [32]. Restoration efforts to improve recruitment conditions, like sand capping or sediment stabilization [35,48], should only be used when variability in recruitment conditions is the main cause of uncertainty.

Action in catchments to improve coastal water quality is an important management option for many coastal ecosystems, especially seagrass [26,45]. Our model found that improving light was beneficial for recovery times across most scenarios for connectivity and causes of uncertainty in recolonization. This result is important because it implies that management can improve connectivity, and hasten natural recovery processes, by mitigating chronic stressors. This result supports findings by others suggesting that mitigating chronic stressors will improve the resilience and recovery of meta-populations of coastal habitats to disturbances [6,50]. More generally, improving water quality will be a safe management option for any sub-tidal coastal habitat, in that it will improve recovery times in most situations. Further, the benefits of improving water quality increased, relative to other management actions, as we approach light levels so low that even plant growth is impossible. However, managers may still elect to focus on direct restoration techniques, like seeding, because of the jurisdictional complexity and time-lags involved with catchment management [51].

We deliberately made several simplifying assumptions in our modelling approach to enhance generality of the findings. Here, we discuss the most important caveats (see full list in electronic supplementary material, table S4).

We found that recovery times, and the benefit of some management actions, were sensitive to the values of parameters for the dispersal and recruitment functions. We set the parameter values by calibrating their values to meet literature expectations for recovery times. Direct field estimates of these parameters would allow estimation of recovery times for particular locations. However, the sensitivity of our modelled recovery times to these parameters is, in itself, helpful; it reveals the importance of quantifying these parameters to accurately estimate recovery times [52]. The modelling also revealed that, under a particular model with a particular level of connectivity, the management action that gave the fastest recovery time was not sensitive to the dispersal and recruitment parameters. This means priorities for restoration action remain the same, even if the expected time to recovery changes. Theoretical studies of marine protected areas have also advanced that field in a similar way, by identifying key uncertainties for management decisions that can become priorities for field measurement [23]. A next step would be to extend our model to cases where uncertainty in recolonization is driven by a mix of recruitment and dispersal processes and also consider optimizing across a mix of management actions. This extension to our model would be straightforward mathematically (sum the uncertain dispersal and recruitment models), and the challenge will be finding sufficient data to parameterize both processes. Data on variation in connectivity [32] and suitability of environmental suitability [42] could be used to determine the relative contributions of dispersal and recruitment uncertainty to recolonization success. Then, the optimal mix of management actions, such as combining sediment stabilization with transplantation [35], could be determined.

Long-term differences in dispersal have implications for genetic diversity of meadows, and genetic diversity can affect long-term survival of populations [53]. Therefore, future models should include a genetic component to better inform restoration actions [33]. We only modelled the effects of the light stressors on biomass growth, and did not consider its effects on propagule survival and dispersal. For instance, environmental stressors may reduce connectivity of animal habitat formers and ability to recover from disturbances [54]. Further study is needed to understand the sensitivity of propagules of marine primary producers to environmental stressors [43]. Finally, for seagrasses, disturbance can increase propagule production [55], whereas we assumed propagule production scaled with seagrass biomass. Thus, for short periods, disturbed meadows could have higher propagule production and connectivity to neighbouring patches. Quantifying the drivers of propagule production will be key to accurate prediction of recovery times, because our sensitivity analysis identified that results were most sensitive to the parameter controlling propagule production.

## 5. Conclusions

We provided a mathematical framework for predicting coastal habitat recovery times following restoration action. Our results help explain why in some circumstances natural recovery has not happened at disturbed seagrass meadows and as such can help set expectations for recovery timescales as well as inform conservation decisions. More generally, this model could be applied to model any connected network of coastal habitats, including coral reefs, mangrove forests, oyster reefs and kelp forests [27,56,57]. Exploring the sensitivity of recovery times in those habitats to parameter uncertainties could reveal key data gaps, as it has done here for seagrass. Drawing on the analogy of marine protected area science, where dynamic models of population processes informed guidelines for the design of protected areas, our model provides a theory to inform guidelines for recovery times of restored coastal habitats. Future extensions to the model can explore recovery timeline for other habitats and the effects of stressors on propagule production.

## Data Availability

R code is available on Zenodo [58]. Supplementary material is available online [59].
